# Come together now: Dynamic body-formation of key regulators integrates environmental cues in plant development

**DOI:** 10.3389/fpls.2022.1052107

**Published:** 2022-11-14

**Authors:** Rebecca C. Burkart, Ali Eljebbawi, Yvonne Stahl

**Affiliations:** ^1^ Institute for Developmental Genetics, Heinrich-Heine University, Düsseldorf, Germany; ^2^ Cluster of Excellence on Plant Sciences (CEPLAS), Heinrich-Heine University, Düsseldorf, Germany

**Keywords:** transcription factor, transcriptional regulator, body-formation, phase separation, plant memory, development, adaptation, environmental stimuli

## Abstract

Plants as sessile organisms are constantly exposed to changing environmental conditions, challenging their growth and development. Indeed, not only above-ground organs but also the underground root system must adapt accordingly. Consequently, plants respond to these constraints at a gene-regulatory level to ensure their survival and well-being through key transcriptional regulators involved in different developmental processes. Recently, intrinsically disordered domains within these regulators are emerging as central nodes necessary not only for interactions with other factors but also for their partitioning into biomolecular condensates, so-called bodies, possibly driven by phase separation. Here, we summarize the current knowledge about body-forming transcriptional regulators important for plant development and highlight their functions in a possible environmental context. In this perspective article, we discuss potential mechanisms for the formation of membrane-less bodies as an efficient and dynamic program needed for the adaptation to external cues with a particular focus on the *Arabidopsis* root. Hereby, we aim to provide a perspective for future research on transcriptional regulators to investigate body formation as an expeditious mechanism of plant-environment interactions.

## Introduction

Plant growth and development are coordinated by a crosstalk between genetically inherited intrinsic signals and environmental extrinsic cues. As plants are sessile, they must adapt to everchanging environments daily, seasonally, and durably. This is important not only for above-ground organs directly exposed to alternating conditions, but also for underground roots, fundamental for anchorage and the acquisition of nutrients and water. However, little is known about how roots perceive, transduce, and respond to environmental stimuli. The balanced homeostasis between maintenance and differentiation of root stem cells (SCs), located in the stem cell niche (SCN) at the root tip, is crucial for root growth and development and requires a robust but dynamic regulation in response to external cues, involving intricate gene regulatory networks (Burkart and Stahl, 2017; Strotmann and Stahl, 2021). Recently, a role of membrane-less bodies containing key transcription factors (TFs) in root SC regulation, has been reported. Here, bodies are discussed to influence SC-fate decisions potentially upon a yet unknown trigger (Burkart et al., 2022), which could provide a link between root growth and environmental influences. The formation of such membrane-less bodies within a liquid environment like the cytosol or nucleoplasm can originate from the separation of biomolecules into two phases. Frequently, both phases are of a liquid-like nature, and the underlying mechanism is hence liquid-liquid-phase-separation (LLPS), but also other states are possible, e.g. gel-like or even solid phases (Alberti et al., 2019).

Other membrane-less bodies were lately reported. They serve as environmental sensors in response to external stimuli like heat, cold, humidity, drought, pathogen interactions, light, day-length, and stress conditions (Zhang et al., 2019; Jung et al., 2020; Dorone et al., 2021; Huang et al., 2021; Wang et al., 2021; Zhu et al., 2021; Zhu et al., 2022). This is crucial for our understanding of how plants adapt to changing environments. However, most of the underlying molecular processes remain elusive, especially in the roots. Although reactions of roots to cold or warm temperatures, factors of the circadian clock, light, nutrient availability, and pathogens have been reported, their fundamental regulation, potentially *via* body-formation, remains largely enigmatic (Plieth et al., 1999; Yazdanbakhsh et al., 2011; Chen et al., 2016; Martins et al., 2017; Li et al., 2019; Gaillochet et al., 2020; Gilbert et al., 2021; Kawa and Brady, 2022; Sharma et al., 2022; Villacampa et al., 2022).

**Figure 1:**
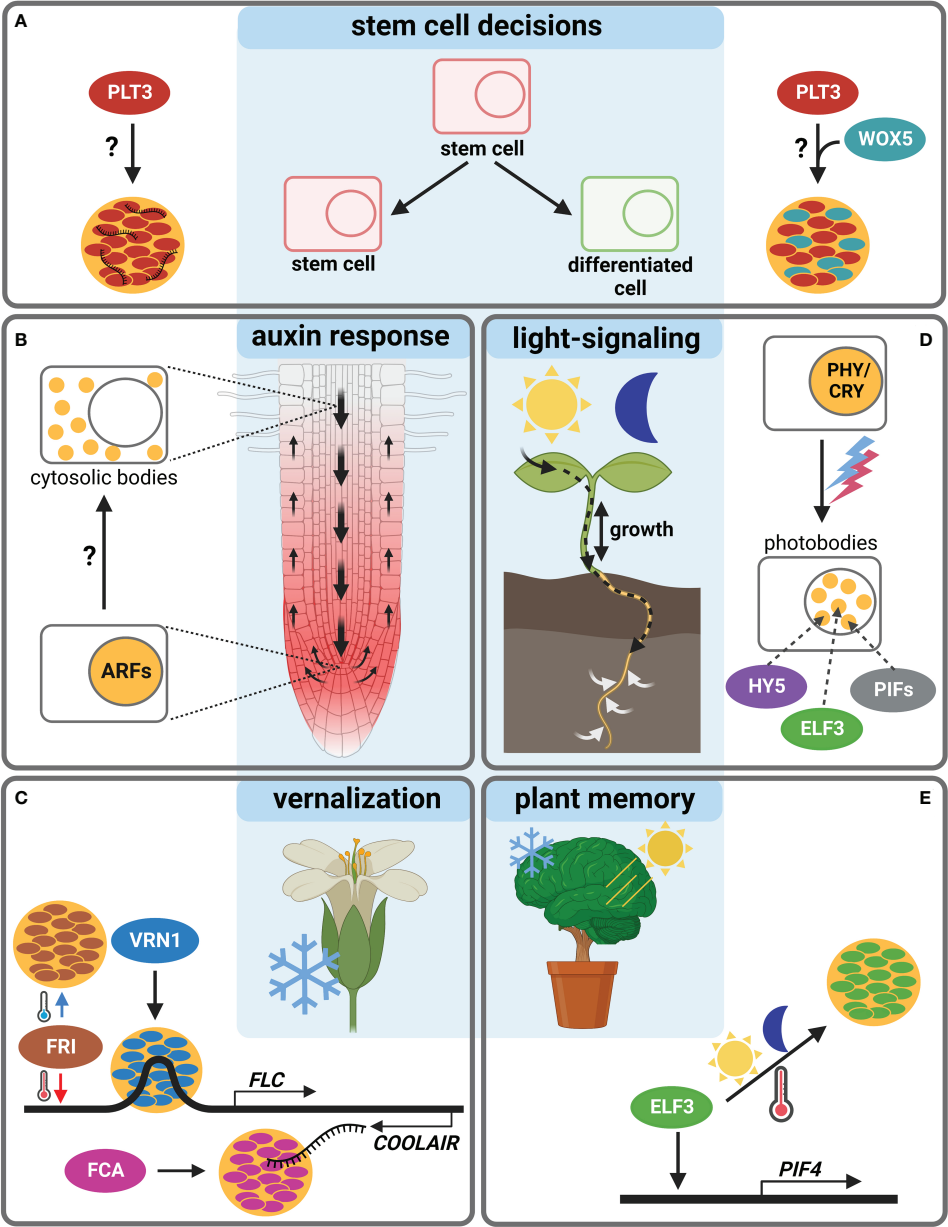
Body-formation of transcriptional regulators during *Arabidopsis thaliana* development. **(A)** The TF PLT3 (red) localizes to NBs (orange circles) in cells undergoing stem cell fate decisions, either with RNA (left) or together with WOX5 (cyan, right). **(B)** ARF TFs form cytosolic bodies (small orange circles) in certain cell types of the Arabidopsis root, initiating a cell type specific auxin response. **(C)** The response to vernalization is regulated by NB formation (orange circles) containing FRI (brown), VRN1 (blue) or FCA (pink). **(D)** Photobodies (small orange circles) form in response to light and contain HY5 (purple), ELF3 (green) and PIFs (grey). **(E)** ELF3 forms NBs (orange circle) in response to warm temperatures and is also involved in circadian clock responses raising the question if this is needed for plant memory. Created with BioRender.com.

In this perspective article, we will highlight examples of body-forming transcriptional regulators (TRs), potentially reacting to environmental changes and discuss their possible roles in *Arabidopsis* development, e.g., in root SCN regulation (**Figure 1** and **Table 1**).

**Table 1:** Body-forming transcriptional regulators in *Arabidopsis thaliana*.

Transcriptional regulator	body-forming domain	phase separation	proposed function	environmental trigger	references
**ARF7, ARF19**	PrD	yes (liquid-to-solid)	sequestration of ARFs for cell-type specific auxin response	?	Powers et al., 2019
**ELF3**	PrDs	yes	switch for activation/ inactivation of ELF3 and short-term memory	warm temperature	Jung et al., 2020; Murcia et al., 2022
**FRI**	IDR and coiled-coil	?	sequestration from FLC-locus	cold temperature	Zhu et al., 2021
**FCA**	PrD	yes	RNA-processing of the FLC antisense transcript COORLAIR	?	Fang et al., 2019
**HY5**	IDR	?	light-controlled switch for gene expression and plant development	light?	Yoon et al., 2006; Ang et al., 1998
**PIFs**	?	?	light-dependent protein degradation; regulation of phyB levels and gene expression	light	Al-Sady et al., 2006; Leivar et al., 2008a, Leivar et al., 2008b
**PLT3**	PrD	?	SC-fate determination	?	Burkart et al., 2022
**VRN1**	IDR	yes	spatiotemporal regulation or super-activation of gene expression	?	Zhou et al., 2019

PrD, prion-like domain; IDR, intrinsically disordered region; ? , unknown/uncertain.

## Protein domains responsible for body-formation

Body-formation often depends on disordered domains in the amino acid sequence of proteins, as reported for PLT3 (Burkart et al., 2022). As the nomenclature found in literature with many ambiguous terms is confusing, an overview of the existing different domains is provided.

Intrinsically disordered regions (IDRs) in proteins are characterized by the lack of a defined 3D-structure, their flexibility, and the potential to switch conformation (Oldfield and Dunker, 2014). They are involved in protein-protein interactions, chromatin organization, gene expression, and LLPS initiation (Oldfield and Dunker, 2014; Cuevas-Velazquez and Dinneny, 2018; Covarrubias et al., 2020; Musselman and Kutateladze, 2021; Shukla et al., 2022). Importantly, IDRs are enriched in eukaryotic TFs, including plants (Liu et al., 2006; Salladini et al., 2020).

Low-complexity domains (LCDs) have a reduced amino acid diversity and are usually, but not exclusively, located inside IDRs. They are intrinsically unfolded in their native state, but also possess structuring effects on IDRs that can be modulated by interaction partners (Romero et al., 2001; Gonçalves-Kulik et al., 2022), and can mediate phase-separation (Molliex et al., 2015).

Prions are proteins that can adopt distinct conformations and convert between different structural and functional states, e.g., self-perpetuation. They are involved in neurodegenerative diseases (Aguzzi et al., 2013), but also have functional roles. Their flexibility facilitates the adaptation to environmental changes, and their self-propagation can act epigenetically to replicate biological information (Shorter and Lindquist, 2005; Chernova et al., 2014; Franzmann et al., 2018). Prionogenic proteins contain “prion-like domains” (PrDs) (Alberti et al., 2009) that mediate phase-separation (Franzmann et al., 2018), which depends on the amino acid composition of the PrD (Bremer et al., 2022).

In the plant kingdom ~500 proteins were identified carrying PrDs (Chakrabortee et al., 2016). Typically, PrDs harbor asparagine (N)- or glutamine (Q)-rich regions that induce protein-aggregation (Michelitsch and Weissman, 2000). Because of the enrichment of certain amino acids in their sequence, PrDs are a subclass of LCDs (Cascarina et al., 2021). However, not all proteins containing polyQs or polyNs display prion-like behavior (Toombs et al., 2010). Like IDRs, also polyN and polyQ tracts are enriched in plant TFs (Kottenhagen et al., 2012). Like prions, polyQ-containing proteins are associated with pathogenic behavior, but also harbor functional traits (Mikecz, 2009), e.g., enhancing the transcription activation potential of TFs (Atanesyan et al., 2012). They are often located within flexible structures lacking resolvable 3D domains, and mediate protein-protein interaction (Totzeck et al., 2017). Like IDRs, LCDs and PrDs, polyQ tracts also mediate phase transitions (Langdon et al., 2018).

## Body-formation of TRs choreographing root development

Recently, a novel model of root SCN regulation was proposed, describing how external signals could be perceived and forwarded, resulting in a reaction of the *Arabidopsis* root (Burkart et al., 2022). Here, a key TF in SCN homeostasis, PLETHORA3 (PLT3), forms PrD-dependent NBs that contain RNA, and are sites for interaction with another important TF, WUSCHEL-RELATED-HOMEOBOX5 (WOX5), pivotal for root SC maintenance (Pi et al., 2015). PLT3-body formation is concentration-dependent and, interestingly, is only observed at distinct timepoints in specific cells undergoing cell fate decisions, e.g., in distal root SCs and young lateral root primordia. Therefore, NB-formation is hypothesized to function as a reversible readout for cell-fate determination, potentially in response to, to date unknown, extrinsic or intrinsic signals (Burkart et al., 2022) (**Figure 1A**).

Furthermore, root TFs of the AUXIN RESPONSE FACTORS (ARFs) family were shown to accumulate cytoplasmic bodies depending on root cell type (ARF7 and ARF19). They contain polyQs in their middle region as well as PrDs necessary for phase-separation, which may act as sequestration sites for cell-type specific auxin responsiveness of ARF proteins (Powers et al., 2019). Yet, a potential environmental trigger for body-formation remains to be determined (**Figure 1B**).

## TR-bodies synchronizing flowering time with the environment

Additional examples of TRs forming membrane-less bodies are mostly involved in the reaction to the environment. The FLOWERING LOCUS C (FLC) is a repressor of floral transition in *Arabidopsis*, downregulated in cold but activated by FRIGIDA (FRI) in warm temperatures. FRI, a major determinant in flowering time, forms cold-induced NBs (Zhu et al., 2021). The FRI-complex binds to the *FLOWERING LOCUS C (FLC)* promoter during warm temperatures, and the NBs sequester FRI away from the *FLC* locus in cold conditions (**Figure 1C**). The FRI-body formation depends on both IDRs and coiled-coil domains. Furthermore, FRI binds to the *COOLAIR* locus located at the 3´-end of *FLC*, which produces an antisense RNA and represses *FLC* in cold conditions. At this site, the FRI binding is stronger at low temperatures, and a *COOLAIR* splicing variant in turn interacts with FRI to promote cold-induced body formation.

Another TR important for vernalization is VERNALIZATION 1 (VRN1) that, like FRI, contains an IDR and undergoes LLPS to form NBs (Zhou et al., 2019). Interestingly, VRN1 binds DNA non-specifically, and the DNA-binding is crucial for phase-separation, leading to DNA-containing bodies. Their function is unknown, but a spatiotemporal regulation of gene expression is essential for the determination of cell fate and identity, and DNA-phase-separation can lead to super activation of gene expression (Sabari et al., 2018). An involvement of environmental signals in VRN1-phase-separation remains unclear, but a temperature-dependency is likely, as VRN1 is involved in vernalization and silences *FLC* in long cold periods (Levy et al., 2002) (**Figure 1C**).

Another prominent example of phase-separation exists at the FLC locus, the alternative 3′-end processing of the *FLC* antisense transcript *COOLAIR*, which requires the RNA-binding protein FLOWERING CONTROL LOCUS A (FCA). FCA phase-separates PrD-dependently, together with other RNA-processing factors, to enhance polyadenylation at specific sites (Fang et al., 2019) (**Figure 1C**). FCA is a TR that reduces transcriptional read-through by promoting proximal polyadenylation and DNA-methylation at many sites in the *Arabidopsis* genome (Sonmez et al., 2011). The environmental influence on FCA-phase-separation is not known, yet a temperature-dependence is also likely, because of the regulation of the *FLC* locus. Consistently, FCA has been shown to mediate thermal adaptation of stem growth through interaction with PHYTOCHROME INTERACTING FACTOR4 (PIF4) (Lee et al., 2014).

## TR bodies in response to light, temperature, and other signals

PIFs form light-dependent bodies (Al-Sady et al., 2006; Leivar et al., 2008a), so-called photobodies, in which phytochrome and cryptochrome photoreceptor families, but also many TRs, e.g., PIFs, EARLY FLOWERING3 (ELF3) and ELONGATED HYPOCOTYL 5 (HY5) light-dependently co-localize (van Buskirk et al., 2012; Wang and Lin, 2020). Photobodies have been proposed to act as sites for protein storage or degradation, transcriptional regulation, component sequestration, and RNA modification (van Buskirk et al., 2012; Ronald and Davis, 2019; Wang et al., 2021). There are recent indications that they are formed by LLPS (Wang et al., 2021), yet their precise composition and function remain obscure. PIFs as key components of photobodies interact with phytochromes (PHYs) and cryptochromes (CRYs) and are important for the nuclear import and accumulation of PHYB (Ni et al., 1999; Pfeiffer et al., 2012; Pedmale et al., 2016). PIFs are basic helix–loop–helix (bHLH) TFs with a PHY-binding motif that regulate the expression of light-responsive genes by transducing light-signals perceived by PHYs or CRYs, thereby regulating plant growth (Martínez-García et al., 2000; Leivar et al., 2008a; Leivar et al., 2008b; Soy et al., 2012; Soy et al., 2014; Ma et al., 2016; Pedmale et al., 2016; Cordeiro et al., 2022). Apart from light, they are proposed to serve as central hubs for integrating diverse external stimuli in *Arabidopsis* and crops, including circadian rhythms, temperature, drought, and salinity (Cordeiro et al., 2022). Besides photoreceptors, PIFs interact with several other factors like ELF3, FCA and ARF6, providing potential links to a plethora of key developmental processes (Lee et al., 2014; Oh et al., 2014; Jiang et al., 2019) (**Figure 1D**).

ELF3, as part of the evening complex, is a key node regulating photosynthesis, circadian clock, and flowering time in addition to diverse signals as temperature and phytohormones (Zagotta et al., 1996; McWatters et al., 2000; Ezer et al., 2017). It acts as a TR repressing *PIF4* and *PIF5* in the evening to regulate hypocotyl growth (Nusinow et al., 2011). Furthermore, ELF3 may link shoot and root responses to external stimuli (Joseph et al., 2015), and it regulates rhythmic root elongation (Yazdanbakhsh et al., 2011), thereby integrating the circadian clock into root growth. Like PLT3, the ELF3 protein contains PrDs including poly-Q tracts important for the perception of environmental cues as they mediate sensitivity for thermal responsiveness and for the temperature-dependent formation of liquid-like bodies, allowing ELF3 to quickly shift between an active and inactive state *via* temperature-dependent phase-transition (Jung et al., 2020) (**Figures 1D, E**).

Similar to ELF3, the bZIP TF HY5 represents an environment-dependent link between shoot and root, as it is a shoot-to root mobile TF regulating shoot growth, carbon assimilation, root growth and nitrogen uptake in response to light (Chen et al., 2016). HY5 has an IDR needed for its interaction with the ubiquitin–protein ligase CONSTITUTIVE PHOTOMORPHOGENIC 1 (COP1) (Yoon et al., 2006) and localizes to COP1-dependent photobodies that may serve as a switch for light-dependent gene regulation (Ang et al., 1998). Furthermore, HY5 was shown to act as a shoot signaling module together with PIFs and phytochromes to control root responses to high ambient temperatures (Gaillochet et al., 2020) (**Figure 1D**).

## Discussion

As IDR- or PrD-containing bodies exist in numerous biological pathways, studying the mechanisms of their formation and, most importantly, their functional relevance is keenly interesting. Here, we summarized the putative roles of plant TR-bodies, spanning SC-regulation, sequestration, spatiotemporal regulation of gene expression, RNA modification, protein degradation, and switches in response to stress, temperature, light or until now unknown triggers.

It is striking that many of the body-forming TRs are involved in diverse pathways with multiple partners and integrated environmental signals. Body-formation could act as a fast and stimulus-dependent switch for spatiotemporal regulation of diverse outputs, e.g., gene expression in response to external cues. A similar spatiotemporal regulation has been discussed for signal transduction of dynamic microdomain-forming receptors at the plasma membrane (Burkart and Stahl, 2017). The most evident example of a body-forming TR that might actively control gene expression is VRN1, as it phase-separates with DNA (Zhou et al., 2019). Phase-separated NBs, which accumulate TFs, enhancers, co-activators and factors of the transcription machinery at high density, may even serve as sites for the super-activation of gene-expression (Sabari et al., 2018).

Furthermore, most TFs contain IDRs or PrDs with polyQs that are often linked to phase-separation (Liu et al., 2006; Cuevas-Velazquez and Dinneny, 2018) and are known to enhance their transcription activation potential (Atanesyan et al., 2012). Consistently, Brodsky et al. (2020) reported that the IDRs located outside of the DNA-binding domains of TFs are necessary and sufficient to find most target-promoters. The IDR-directed binding-specificity depends on low-affinity interactions between IDR and DNA. The target-promoter will be found by a two-step-process where the IDR first localizes the TF to a broad DNA-region and then the DNA-binding domain finds the precise binding site. This two-step model was further adapted suggesting that the IDRs mediate protein-protein interaction, bringing together several factors that search and bind the target-sites specifically (Staller, 2022).

Additionally, DNA-containing bodies could also act as chromatin-remodeling-sites. Supporting this, VRN1 silences *FLC* during long cold periods through chromatin-modifications (Levy et al., 2002; Bastow et al., 2004; Mylne et al., 2006), possibly in DNA-containing VRN1-bodies. Moreover, FRI associates with many regulators of the *FLC*-locus, including TFs and chromatin modification factors, to control the transcription of *FLC* (Choi et al., 2011). In addition to chromatin-modification, the association of FRI with TFs also supports the above-mentioned two-step model of TF-interaction and subsequent DNA-target-binding (Staller, 2022), indicating that several processes could overlap and synergize. Furthermore, cell-type-specific chromatin-modeling on a submegabase scale regulates the cell fate of mammalian SCs (Phillips-Cremins et al., 2013), and could similarly influence plant SC fate, e.g., PLT3-dependently in distal root SCs. A role in chromatin-remodeling has also been reported for PIF-regulated genes leading to fast, light-controlled transcriptional induction (González-Grandío et al., 2022).

Moreover, environmentally induced VRN1-dependent epigenetic chromatin-silencing was shown to be mitotically but not meiotically stable (Mylne et al., 2006). Thereby, biological information about vernalization will be inherited by the next generation of cells and throughout the rest of the plant’s life but will be reset for the next plant generation. Thus, VRN1 contributes to a cold-induced memory of winter. Since the chromatin-modification could happen in DNA-containing bodies, they may play a critical role in plant memory. A vernalization-induced VRN1-dependent histone-methylation within the *FLC* locus has been reported earlier and was proposed to act as an epigenetic memory of winter (Bastow et al., 2004).

The role of TF-bodies as memory of certain environmental cues is intriguing, as plant life strongly depends on its surroundings and on the anticipation of rhythmic environmental changes. Therefore, memory plays a crucial role in optimal adaptation. The role of prions as protein-based memory is an attractive emerging theory. Prions can self-propagate conformations that are epigenetically stable, allowing the storage of biological information that can even be inherited. Prions are debated as basis for long-term memory (LTM) in humans (Sudhakaran and Ramaswami, 2017). Here, RNA-binding proteins with PrDs enable the storage of mRNA needed for LTM and even the formation of RiboNucleoProtein granules *via* phase-separation, mediated by LCDs, is proposed. Functional prion-proteins are discussed to convert to an insoluble form upon a physiological trigger and that their subsequent self-perpetuation has a physiological function, e.g., maintaining long-term changes in synaptic efficacy, contributing to LTM (Si and Kandel, 2016).

A similar function of prion-proteins in plant memory is intriguing because of their ability to react to previous physiological conditions. Recently, the response of the plant not only to current but also to preceding temperatures was reported, thereby providing a short-term memory for previous conditions. Here, the day-time temperature affects nuclear PIF4 and HY5 levels during the next night. This process requires ELF3 which is sequestered into phase-separated bodies. The ELF3 concentration and the capability of body-formation vary during the day-night cycle, depending also on the previous night-time temperature. Warm night-time temperatures promote the formation of ELF3-NBs in hypocotyl cells during the afternoon but not in the morning. The formation of ELF3-bodies shows hysteresis, as the sensitivity to cooling shifts significantly compared to warming. *PIF4* promoter activity correlates with ELF3 body-formation, as ELF3 achieves hysteresis and drives the *PIF4* promoter into the same behavior, thus setting a memory of preceding temperature-conditions (Murcia et al., 2022) (**Figure 1E**). This aspect could be investigated further, as PIF4 and HY5 are additional regulators in light-signaling. The temperature-dependent variation of nuclear PIF4 and HY5-concentrations could therefore influence their capability of possibly light-dependent photobody-formation, thereby regulating the light-response mechanism in dependence of the memorized daytime-temperature. In this case, the formation of bodies would serve as protein-based and environment-dependent memory. It remains to be elucidated if not only temperature, but also other environmental cues could cause similar hysteretic effects in body-formation, thereby serving as a plant memory for diverse external influences.

Like the shoot, also the plant root is exposed to environmental changes and rhythms and thus also needs a mechanism to memorize. The root SCN is essential for efficient plant growth and development, which relies on the delicate balance of SC maintenance and differentiation. This could be optimized by the plant in response to memorized environmental conditions. The observed body-formation of PLT3 could represent such a mechanism, thereby driving SC fate in the needed direction. However, further research is needed to confirm this hypothesis. Finally, we propose that dynamic TR-body formation could represent a fast and reversible switch to respond to current external stimuli, important for the plant’s immediate reaction to the environment, or serve as memory for later adaptation of plant growth and development. Further research is needed to understand not only the molecular mechanism responsible for dynamic body-formation in response to differential environmental cues, but also set their physiological context associated with plant growth and development.

## Data availability statement

The original contributions presented in the study are included in the article. Further inquiries can be directed to the corresponding author.

## Author contributions

RB conceptualized and wrote the article. YS conceptualized and edited the article. AE helped editing the article. All authors contributed to the article and approved the submitted version.

## Funding

This work was supported by the Deutsche Forschungsgemeinschaft (DFG) *via* grants STA 1212/1-1 and STA 1212/6-1 to YS.

## Conflict of interest

The authors declare that the research was conducted in the absence of any commercial or financial relationships that could be construed as a potential conflict of interest.

## Publisher’s note

All claims expressed in this article are solely those of the authors and do not necessarily represent those of their affiliated organizations, or those of the publisher, the editors and the reviewers. Any product that may be evaluated in this article, or claim that may be made by its manufacturer, is not guaranteed or endorsed by the publisher.
